# Haematology and Serum Biochemical Indices of Lambs Supplemented with *Moringa oleifera*, *Jatropha curcas* and *Aloe vera* Leaf Extract as Anti-Methanogenic Additives

**DOI:** 10.3390/antibiotics9090601

**Published:** 2020-09-14

**Authors:** Abiodun Mayowa Akanmu, Abubeker Hassen, Festus Adeyemi Adejoro

**Affiliations:** Department of Animal Science, University of Pretoria, Private bag X20, Hatfield 0028, Pretoria, South Africa; abubeker.hassen@up.ac.za (A.H.); festus.adejoro@tuks.co.za (F.A.A.)

**Keywords:** antinutritional, haematology, medicinal plant, Merino sheep, toxicity

## Abstract

Medicinal plants have been found to be effective in a wide range of applications in ruminant animals. However, some plant extracts may be toxic to animals, depending on their seconday metabolite composition and dose, and therefore, animal trials are needed to validate their safety when used as anti-methanogenic additives. This study investigated the effect of three plant extracts used as anti-methanogenic dietary additives, on the haematology and serum biochemical parameters in sheep. Methanolic extracts of *Moringa oleifera* (MO), *Jatropha curcas* (JC) and *Aloe vera* (AV) were orally dosed as experimental treatments for 75 days to sheep, and their effect on the haematology and serum biochemical parameters of SA Mutton Merino (SAMM) lambs were compared with sheep on a control treatment without any additive treatment. Extracts of MO, JC and AV were extracted in 100% methanol, freeze-dried, and reconstituted in distilled water. A total of 40 lambs were ranked according to their body weight into a group of four and one sheep at a time was randomly allocated into four dietary treatments which include a control treatment, and treatment with either MO, JC or AV extract. Lambs were drenched twice daily with doses equivalent to 50 mg/kg dry matter intake (DMI) based on previous week feed consumption. Blood samples were collected via jugular vein puncture and analysed for haematology and serum biochemistry parameters, using standard procedures. The results of the haematological analysis showed that most haematological parameters were not affected by plant extract used as anti-methanogenic additives (*p* > 0.05), except for higher white blood cell (WBC) and lymphocytes counts recorded in control lambs and lambs in the AV treatment. All serum biochemical properties (except alkaline phosphatase) were not different (*p* > 0.05) between the control and lambs treated with plant extracts. Alkaline phosphatase was influenced by the plant extract (*p* < 0.05), with lambs receiving MO, JC and AV having lower alkaline phosphatase concentrations compared to lambs on the control diet without any additive. The result of the study showed that extracts of MO, JC and AV were not toxic to sheep when used as antimethanogenic additives at the recommended dose of 50 mg/kg dry matter feed which had proved previously to be effective in reducing enteric methane emission. Therefore, these plant extracts could be used safely as alternative dietary additives to reduce enteric methane emission and boost the productivity of SA Mutton Merino sheep.

## 1. Introduction

The use of medicinal plants as dietary additives is gaining increasing research interest due to the wide biological diversity and potential beneficial effects for sustainable livestock production [1]. The plant secondary metabolites (PSMs) (also called phytonutrients) inherent in these medicinal plants have been found to exhibit strong antibacterial and antifungal properties, resulting in strong antimethanogenic properties in ruminant animals among other benefits reported in different studies [1,2]. Equally, some medicinal plants and their extracts tend to improve nutrient digestibility in ruminant livestock [3]. Previous studies showed that extracts of *Moringa oleifera* (MO), *Jatropha curcas* (JC) and *Aloe vera* (AV) significantly reduced in vitro methane production when supplemented to a typical ruminant diet [2]. Moringa is reported to contain a moderate concentration of flavonoids, alkaloid and only negligible concentration of tannins [4], while Jatropha contains a high concentration of phorbol esters [5,6].

However, while some of these PSMs are capable of binding to specific receptors in neurons, intestines, and other cells and exhibit favourable physiological effects [7], others may be antinutritional and exert toxic effects on animals consuming them, depending on the type of plant or parts, or amount consumed [6,8]. Even though the use of methanolic extracts of Moringa, Jatropha and *Aloe vera* has been validated in vitro [2,9], nevertheless, a great deal of consideration should be given to the impact of these ‘natural alternatives’ on the health of animals before being recommended for wider application. For example, the phorbol esters in *Jatropha curcas* at a concentration above 1.45 mg/kg body weight (BW) in ruminants were found to exert reduced performance, physiological toxic effects and cause eventual death [5,10,11]. Equally, high doses of *Aloe vera* were associated with diarrhoea, kidney failure, phytotoxicity and hypersensitive reactions in some animal species [12] and this was associated with the inherent anthraquinones and phenolic compounds. In contrast, previous studies on the feeding of moringa leaves to goats did not report any toxicity symptoms [13].

Under research scenarios, the use of extracts helps to narrow down the direct impact of PSMs on the biological activity and dose of extract without the confounding effects associated with dietary characteristics of plant materials or whole plants. Moreover, some structure of plants like the barks of trees are difficult to feed to animals but also contain valuable phytochemicals. Previous in vitro and in vivo trials have validated the anti-methanogenic effects of MO, JC and AV when used as dietary additives at 50 mg/kg DM of substrate [2,9,14], however, potential harmful effects on the animals were not established [14]. To justify this previous in vitro trial as well as make wider recommendations on the beneficial effects of extracts of MO, JC and AV in methane reduction and improvement in feed digestibility, there is a need to evaluate the potential impact of these medicinal plant extracts on the physiology of the animals. This study, therefore, tested the effect of MO, JC and AV extract used as antimethanogenic additives on the haematology and blood chemistry of South African Mutton Merino (SAMM) lambs.

## 2. Materials and Methods

### 2.1. Study Area, Experimental Design and Preparation of Plant Extracts

The study was conducted at the University of Pretoria Experimental Farm, Hatfield, South Africa. The annual rainfall in Pretoria is about 573 mm and the city is located at 1700 m above sea level. This study was approved by the Animal Ethics Committee of the University of Pretoria with approval number ECO-030-14. Fresh foliage of MO, JC and AV was collected, freeze-dried and the extract obtained using 100% methanol as reported previously [2]. Extract solutions were prepared from an equal mass of each plant material by solubilising 100 mg of dried plant extracts in 100 mL of distilled water, stirring with an overhead stirrer and stored as a stock solution. Extracts solutions were administered at a dosage of 50 mg/kg of feed DM consumed while the actual dosage drenched to each animal was adjusted weekly using the previous week’s feed intake. The 50 mg/kg dry matter intake (DMI) dose of extract was based on previous in vitro trials which established the effective dose at which the extracts reduced enteric methane [2,9,14]. Forty 4-month-old SAMM ram lambs with an average live weight of 28.8 ± 0.40 kg were blocked according to their body weight, and from each block randomly allocated into four dietary treatments which include (i) total mixed ration diet (TMR) + distilled water as a control, (ii) TMR + MO extract (50 mg/kg DMI), (iii) TMR + JC extract (50 mg/kg DMI) and, (iv) TMR + AV extract (50 mg/kg DMI). The lambs were housed in open-sided pens with concrete floors and metal roofing. Water was provided ad lib while routine vaccination and prophylactic treatments were carried out prior to the start of the trial. Two lambs from each treatment within a block were housed in a pen with five pens per treatment and a total of ten lambs per treatment.

### 2.2. Experimental Diet, Blood Sample Collection and Analysis

The ingredient and nutrient composition of the experimental diet is shown in Table 1 and contains 42% roughage and 58% concentrate. Lambs were initially adapted to the standard total mixed ration for fourteen days, followed by an additional twelve-day adaptation period, where they received an incremental dosage of the extracts, starting from 10 mg/kg DMI up to 50 mg/kg DMI with 10 mg/kg DMI increment every 3 days to acclimatize lambs to the plant extracts. During this period, lambs were monitored closely to observe any clinical signs of toxicity such as frothing at the mouth, diarrhoea, dehydration, bloody stool or sudden decline in feed consumption. Growth performance of lambs was monitored weekly and feed intake monitored daily for further 75 days after adaptation to feed and extract drenching. Extract drenching did not affect the average daily gain in lambs (average of 270 g/day) while DMI was equally not affected by extract supplementation with lambs consuming 1200 g/day and receiving an average of 60 mg extract solution/animal/day over the study period [14].

Lambs received the oral doses of plant extracts continuously for 75 days during which daily feed intake and weekly body weight changes were monitored. At the end of the trial period, blood samples were drawn from all the forty animals via the jugular vein puncture. Five mL blood samples were collected into BD vacutainer tubes (BD-Plymouth UK), one which contained ethylene diamine tetra-acetic acid (EDTA) for haematological analysis and another without EDTA for biochemical analyses which include the concentration of urea nitrogen, glucose, cholesterol, total serum protein, albumin, globulin, aspartate transaminase (AST), alanine transaminase (ALT), and alkaline phosphatase (ALP). The samples were immediately transferred to the Veterinary Diagnostic Laboratory, Faculty of Veterinary Science, University of Pretoria, Onderstepoort, South Africa, for processing and analysis. Complete blood analysis was done using a multi-parameter automated haematology analyser (ADVIA 2120i, Siemens, South Africa) and blood chemistry was analysed using the Cobas Integra 400 Plus (Roche, South Africa).

### 2.3. Statistical Analyses

Data obtained from this study were analysed using the general linear model procedure of SAS 9.4 (SAS Institute Inc., Cary, NC, USA). The statistical model included treatment effect, block and random error as follows:

y_ijk_ = μ + Block + P_i_ + ε_ijk_ (1)

where y_ijk_ = observation ‘k’ at four extract P (i; control, MO, JC, and AV); μ = overall mean; Block = effect of blocking, P_i_ = plant extracts; and ε_ijk_ is the effect of random error. Where F-test revealed significant difference, means were separated using the Tukey test.

## 3. Results and Discussion

Results show that the haemoglobin levels, red blood cell (RBC) number, haematocrit, mean corpuscular volume (MCV), mean corpuscular haemoglobin (MCH) content, mean corpuscular haemoglobin concentration (MCHC), monocytes, eosinophil and platelet counts was not affected (*p* > 0.05) by supplementation of MO, JC and AV extracts (Table 2). While MCHC estimates the amount of haemoglobin per unit volume in a single red blood cell by taking into account the volume or size of the red blood cell, MCH estimates the proportion of haemoglobin to red blood cells in a given volume of blood. White blood cell (WBC) and lymphocyte count were affected by medicinal extract supplementation (*p* < 0.05) with lambs consuming MO and JC extracts having lower WBC and lymphocyte counts compared to lambs receiving the control or AV extracts. Nevertheless, the observed values for all the haematological values were within the range reported for Merino lambs [15].

Due to their wide biological activity, medicinal plants are widely used in curative therapy involving human and animal subjects. Plant secondary metabolites were reported to regulate immune cells related to adaptive and innate immunity in challenged or non-challenged cows [7]. The immunomodulatory activity of many PSMs has been exploited in veterinary disease management [16]. For example, a methanolic extract of Jatropha leaf at 33.62 mg/mL significantly inhibited influenza A (H1N1) virus replication without any toxicity effect on kidney cells in vitro [17]. While elevated WBC or lymphocyte count may be indicative of viral, fungal or bacterial infection, decreased lymphocyte levels may also indicate a state of compromised immunity such as with reduced T-helper and CD4+ T cell numbers associated with immune suppression in animals [7]. Lymphocytes are a component of the WBC, responsible for both humoral and cellular immunity. In the study by Amirghofran et al. [16], extracts of *Linum persicum* resulted in a significant decrease in lymphocyte proliferation in humans. Contrary to the response observed for MO extract in the study by Jiwuba et al. [18], Moringa supplementation resulted in increased WBC count but had no effect on lymphocyte count in West African Dwarf goats. Equally, goats receiving 10 mL of 4% *Aloe vera* extract solution showed a decline in WBC count but not in lymphocyte count [19]. Nevertheless, the values across the lambs were within the range of 2.1–10.2 × 10^9^/L in lymphocytes and 5.1–15.9 × 10^9^/L in WBC count, normal ranges reported by Lepherd in the literature [15], thus ruling out leucocytosis.

Contrary to the observed responses in this study, previous studies with feeding Jatropha showed significant compromises in animal health indicators. Goats, sheep or calves force-fed with Jatropha seed meal or leaf meal had decreased haemoglobin, PCV, and RBC due to haemoconcentration and dehydration which culminated in cytological damage and the eventual death of animals [20,21]. Doses used in those studies ranged from 0.25–10 g/kg/d. The toxicity effects of Jatropha have been noted to vary with the parts of the plant used, the concentration of extract, the mode of administration, and the organism consuming it [22].

Blood urea nitrogen values are used to determine kidney functionality because urea is a waste product, which is removed during glomerular filtration unless re-utilised in the rumen via the urea cycle. Furthermore, ruminal fermentation ensures that little or no glucose originates from dietary carbohydrate but through hepatic glycogenolysis and therefore, serum glucose concentration may be indicative of liver function in ruminants [23]. Higher levels of AST, ALT and ALP above 140 U/L, 45 U/L and 464 U/L, respectively, could signal hepatotoxicity because they are specific liver enzymes [7,15]. Supplementation of SAMM lambs with Moringa, Jatropha, and *Aloe vera* leaf extract did not affect blood urea nitrogen (BUN) glucose, cholesterol, total serum protein, albumin, globulin, aspartate transaminase (AST) and alanine transaminase (ALT) concentration in the serum (Table 3). However, alkaline phosphatase (ALP) concentration differed among the lambs (*p* < 0.05) with lambs receiving Moringa, Jatropha and *Aloe vera* having lower ALP compared to lambs in the control group. Nevertheless, the concentration of these enzymes is indicative that no significant metabolic disorder occurred in the lambs.

Serum enzymes are indicators of feed quality as they help to detect abnormal changes in response to drugs and phytonutrients long before the death of an animal. The concentrations of total serum protein and cholesterol in the blood are regulated to balance physiological functions that cater for immunity, coagulation, small molecule transport and inflammation, and any huge variation in the concentration of these serum variables might indicate impaired physiological function [24]. While PSMs like garlic extract have shown potential to ameliorate the damage caused by factors inducing oxidative stress indicators [25], studies on Jatropha seeds or leaves in goats, sheep or calves at concentrations ranging from 0.25–10 g/kg/d showed negative responses such as decreased glucose levels, increased serum arginase and glutamate oxaloacetate transaminase activities, increase in serum aspartate aminotransferase (AST) activity, increase in urea concentration, and decrease in total protein and albumin levels, all reflecting significant hepato-renal damage [20,21]. In our study, however, none of these tested parameters were affected by JC extract. In contrast to the finding of this study for MO extract, Moringa leaf meal supplementation reduced creatinine concentration in West African Dwarf goats but did not affect ALP, AST and ALT concentrations [18].

## 4. Conclusions

The leaf extracts of *Moringa oleifera*, *Jatropha curcas* and *Aloe vera* tested as anti-methanogenic additives at 50 mg/ kg DM intake did not elicit any negative effect on the blood profile of the lambs, parameters that are indicative of the health status of the lambs. This means that their use as a dietary anti-methanogenic additive is justified.

## Figures and Tables

**Table 1:** Composition and chemical analysis of total mixed ration fed to SA Mutton Merino sheep receiving various plant extract dosages.

Parameter	Composition
Ingredient (g/kg)	
Soybean meal	170
Yellow maize	280
Alfalfa hay	200
*Eragrostis curvula* hay	222
Molasses	60.0
Wheat offal	50.0
Urea	8.00
Vitamin-mineral premix *	5.00
Total volume	100
Chemical composition (g/kg Dry matter)	
Crude protein	183
Starch	181
Neutral Detergent Fibre	345
Acid Detergent Fibre	206
Acid Detergent Lignin	245
Ash	64.0
Metabolisable Energy (MJ/kg DM)	9.1

* Premix contains in g/kg the following: vit A, 18,000 iu; vit D, 3920 iu; vit E, 2.45 iu; Zn, 5.0 mg; Mn, 4.1 mg; Cu, 0.5 mg; Se, 0.2 mg; Mg, 28 mg; and Co, 0.3 mg.

**Table 2:** Haematological parameters of SA Mutton Merino lambs drenched for 75 days with extracts of *Moringa oleifera*, *Jatropha curcas* and *Aloe vera.*

Parameters	Control	*M. oleifera*	*J. curcas*	*A. vera*	SEM	*p* Value
Haemoglobin (g/L)	120.4	120.1	119.8	115.5	8.31	0.1254
Red blood cells (H × 10^12^/L)	11.67	11.18	11.27	11.07	1.02	0.0911
White blood cells (×10^9^/L)	8.42 ^a^	6.78 ^bc^	5.99 ^c^	8.21 ^ab^	1.59	0.0145
Haematocrit (L/L)	0.35	0.34	0.34	0.34	0.02	0.0751
MCV (fL)	30.15	30.94	30.49	30.91	2.11	0.1112
MCH (pg)	10.49	10.87	10.75	10.62	0.81	0.4785
MCHC (g/dL)	34.77	35.15	35.28	34.42	1.39	0.9652
Red cell distribution (%)	17.71	17.78	17.92	17.41	1.34	0.7541
Segmented neutrophil (×10^9^/L)	3.39	3.03	2.67	3.04	1.16	0.2532
Lymphocytes (×10^9^/L)	4.73 ^a^	3.48 ^b^	3.36 ^b^	4.75 ^a^	1.02	0.0235
Monocyte (×10^9^/L)	2.22	4.36	2.11	3.10	1.71	0.0652
Eosinophil (×10^9^/L)	0.05	0.06	0.05	0.07	0.02	0.9251
Basophil (×10^9^/L)	0.02	0.00	0.00	0.00	0.02	0.2411
Platelet count (×10^9^/L)	508.2	628.2	636.0	575.6	211	0.5521

MCV: mean corpuscular volume; MCH: mean corpuscular haemoglobin; MCHC: mean corpuscular haemoglobin concentration. ^abc^: Means with different superscript across the rows differed significantly (*p* < 0.05).

**Table 3:** Blood biochemical indices of SA Mutton Merino sheep drenched for 75 days with extracts of *Moringa oleifera*, *Jatropha curcas* and *Aloe vera*.

Parameters	Control	*M. oleifera*	*J. curcas*	*A. vera*	SEM	*p*-Value
Urea nitrogen (H mmol/L)	8.78	9.41	9.04	10.02	1.57	0.343
Glucose (H mmol/L)	3.33	3.42	3.39	3.25	0.39	0.785
Cholesterol (mmol/L)	1.42	1.47	1.53	1.49	0.32	0.896
Total serum protein (g/L)	66.58	64.07	64.31	65.6	4.31	0.544
Albumin (g/L)	36.58	37.58	35.06	36.68	2.81	0.291
Globulin (g/L)	30	26.48	29.24	28.92	3.66	0.311
AST (U/L)	106.7	113.1	152.7	114.1	61.3	0.371
ALT (U/L)	14.58	14.55	22.74	15.63	4.17	0.549
ALP (U/L)	335.6 ^a^	259.6 ^b^	267.3 ^b^	221.1 ^b^	10.2	0.047

AST: aspartate transaminase; ALT: alanine transaminase; ALP: alkaline phosphatase. ^ab^: Means with different superscript across the rows differed significantly (*p* < 0.05)
